# Association between Particulate Matter Pollution Concentration and Hospital Admissions for Hypertension in Ganzhou, China

**DOI:** 10.1155/2022/7413115

**Published:** 2022-02-17

**Authors:** Chenwei Li, Xinye Zhou, Kun Huang, Xiaokang Zhang, Yanfang Gao

**Affiliations:** School of Public Health and Health Management, Gannan Medical University, Jiangxi 34100, China

## Abstract

Fine particulate matter (PM_2.5_) and respirable particulate matter (PM_10_) are two major air pollutants with toxic effects on the cardiovascular system. Hypertension, as a chronic noncommunicable cardiovascular disease, is also a risk factor for several diseases. We applied generalized linear models with a quasi-Poisson link to assess the effect of air pollution exposure on the number of daily admissions for patients with hypertension. In addition, we established a two-pollutant model to evaluate PM_2.5_ and PM_10_ hazard effect stability by adjusting the other gaseous pollutants. Results showed that during the study period, 24 h mean concentrations of ambient PM_2.5_ and PM_10_ at 38.17 and 59.84 *μ*g/m^3^, respectively, and a total of 2,611 hypertension hospital admissions were recorded. Air pollution concentrations significantly affected the number of hospitalizations for hypertension approximately 2 months after exposure. For each 10 *μ*g/m^3^ increase in PM_2.5_ and PM_10_ in single-pollutant models, the number of hospitalizations for hypertension increased by 7.92% (95% CI: 5.48% to 10.42%) and 4.46% (95% CI: 2.86% to 5.65%), respectively, at the lag day with the strongest effect. NO_2_, O_3_, CO, and SO_2_ had different significant effects on the number of hospitalizations over the same time period, and PM_2.5_ and PM_10_ still showed robust significant effects after adjustment of gas pollutants through a two-pollutant model. These findings may contribute to a better understanding of the health effects of ambient particulate matter.

## 1. Introduction

Environmental pollution is a leading cause of premature death and disability in the world at present. Against the background of widespread problems in the world, the development of urbanization has made air pollution exposure more prominent in its harmfulness, universality, and persistence. As a result, air pollution has become an environmental risk factor that greatly affects health, leading to a large economic burden of diseases to society [1]. Approximately 7.8 million cardiovascular and respiratory disease hospital admissions were attributed to short-term exposure to all air pollutants. The economic loss of the overall health burden (premature mortality and hospital admissions) was 2,065.54 billion yuan, which was equivalent to 2.5% of the national GDP in 2017 [2]. Among the air pollutants, ambient particulate matters (fine particulate matter with an aerodynamic diameter <2.5 *μ*m (PM_2.5_) and inhalable particulate matter with an aerodynamic diameter <10 *μ*m (PM_10_]) have been widely regarded as important toxic components of air pollution mixtures [3]. A study about the association between ambient fine particulate pollution and hospital admissions for cause-specific cardiovascular disease suggests that in China, short-term exposure to PM_2.5_ is associated with increased hospital admissions for all major cardiovascular diseases except for hemorrhagic stroke, even for exposure levels not exceeding the current regulatory limits [4]. Another study found that the effects of atmospheric PM_10_ on the years of life lost of the three diseases were statistically significant on the different lag days (all *P* < 0.01) in Tianjin, and the maximum effect of PM_10_ appeared in Lag01. The effects from the largest to the lowest were 2.86 (95% CI: 1.79–3.93) person-years for cardiovascular system diseases, 1.59 (95% CI: 0.95–2.23) person-years for ischemic heart diseases, and 1.07 (95% CI: 0.43–1.71) person-years for cerebrovascular diseases [5].

Hypertension, as the major risk factor for cardiovascular disease, not only brings the great economic burden of disease to society but also seriously endangers people's quality of life and life safety [6–9]. A large body of epidemiological and clinical research has shown that exposure to ambient air pollution increases the risk of hypertension [10]. A meta-analysis of epidemiological investigates the associations of short- and long-term exposure to ambient air pollutants with hypertension. These results suggest that short- or long-term exposure to some air pollutants may increase the risk of hypertension [11].

The impact levels of air pollution on specific cities have not been fully reported, particularly Ganzhou City in Jiangxi province. Ganzhou City is located in the south-central subtropical region, showing a typical subtropical monsoon wet climate with prevailing winter/summer wind, spring/summer precipitation concentration, milder climate, abundant heat, full rainfall, heat and cold currents with short duration, and long frost-free period. The demographics up to July 2019 show that Ganzhou City has a resident population of 86,565,000 people; 14.5% of the population includes over 60 years old. The city has a total area of 39,379.64 km^2^, making it a base for the rare metal industry in China. The main sources of local air pollution are automobile exhaust pollution and combustion and dust raised by production construction.

We used the generalized additive model of Poisson regression to analyze the relationship between the time series of air pollution concentration changes in the Ganzhou central area and the number of hypertensive inpatients to explore the risk of exposure to major air pollutants on hypertension.

## 2. Methods

### 2.1. Hypertension Hospitalization Data

Records from hospital admissions for cardiovascular disease (CVD) between 1 January 2016 and 31 December 2020 were extracted from the Big Data Center of the First Affiliated Hospital of Gannan Medical University. We extracted information on the patient's date of hospital admission, principal diagnosis, age, and sex from each hospital admission record. According to the International Classification of Diseases, 10th Revision (ICD-10) codes: hypertension (ICD-10: I10–I13), we screened out discharged patients with a primary diagnosis of hypertension and calculated the number of daily hypertension admissions from 2016 to 2020 using the Excel frequency formula.

This study does not involve experimental animals or individual information on human subjects. Ethical approval was not required for this study. Research projects that do not involve human participants, their data, or tissues do not require ethical review by the ethics committee in Gannan Medical University.

### 2.2. Air Pollution and Meteorological Data

First, we downloaded and collated real-time air pollutant concentrations and meteorological data for 1 January 2016 and 31 December 2020, respectively, from the National Urban Air Quality Real-Time Publishing Platform and the US National Climate Data Center. Then, we calculated the 24-hour mean concentrations of five air pollutants, including PM_2.5_ and PM_10_, carbon monoxide (CO), sulfur dioxide (SO_2_), nitro carbon dioxide (NO_2_), and the daily maximum 8 h mean ozone concentration (O_3_). In addition, the average daily temperature (°C) and relative humidity (%) over the same period were collated.

### 2.3. Statistical Analysis

Daily hospitalizations for hypertension, air pollution, and meteorological data were described as mean, standard deviation (SD), maximum value, and minimum value. *P* < 0.05 was considered statistically significant. All descriptive analyses were performed using the SPSS software. Kruskal–Wallis test was applied to compare the difference in air pollution concentrations in different years [12]. Spearman's correlation was used to estimate the associations between air pollutants and meteorological conditions [13].

The daily hypertension hospitalization data analyzed in the study from 2016 to 2020 are small probability events occurring in a long time series. They belong to qualitative data, conform to the Poisson distribution, and adopt the generalized linear model with Poisson linkage [14]. The generalized linear model of Poisson distribution was established to estimate the effects of air pollutants on hypertension patients. Health outcome variables, air pollutant variables, and meteorological factors should be included in the model. Time trend day of the week (DOW) was generated from the hospitalization dates of hypertensive patients to generate their single-day lag or moving average lag sequence from air pollutant concentration variables. In accordance with previous studies, we used 7 degrees of freedom (df) per year for the smooth function of calendar time to control hospitalization fluctuations on long time trend, 6 df for the current day's temperature, and 3 df for relative humidity [15,16]. We first used a single-pollutant model to examine the independent association between air pollutants and the risk of exacerbating hypertension.

Single-pollutant model:

(1) $$\begin{array}{c} \text{Log}E\left(Y_{t}\right)=\beta Z_{t}+ns\left(\text{time},df\right)+ns\left(X_{i},df\right)+\text{factor}\left(DO\;W_{t}\right)+\mathrm{int}\text{ercpt,} \end{array}$$

where log*E* (*Y*_*t*_) stands for the expected number of hypertension hospital admissions on day *t*; *Z*_*t*_ indicates the concentrations of pollutants (SO_2_, PM_10_, PM_2.5_, NO_2_, CO, and O_3_); *β* stands for the coefficient for *Z*_*t*_; ns( ) represents a smoother based on adjusted smoothing splines, which capture the nonlinear relationships of the covariates of time trend, temperature, and humidity with daily hospitalization; and df represents the degree of freedom.

### 2.4. Sensitivity Analysis

We also conducted sensitivity analyses to check the robustness of results by changing the df in the smooth function of time trend, temperature, and relative humidity. Then, a two-pollutant model containing another pollutant was constructed to study the association of PM_2.5_ and PM_10_ with the number of hospitalizations for hypertension after adjusting the effects of other gaseous pollutants. The robustness of the hazardous effects of particulate matter is verified, confirming that significant results are unaffected by the collinearity of the associated stronger pollutants.

Two-pollutant model:

(2) $$\begin{array}{c} \text{Log}E\left(Y_{t}\right)=\beta_{1}Z_{t}+\beta_{2}K_{t}+ns\left(\text{time},df\right)+ns\left(X_{i},df\right)+\text{factor}\left(DO\;W_{t}\right)+\text{intercpt,} \end{array}$$

where *Z*_*t*_ indicates the concentrations of the main effect of air pollutants to be calculated in the model (PM_2.5_ or PM_10_), *K*_*t*_ is the concentration of air pollutants affecting the hypertension hospital admissions under the combined action of *Z*_*t*_, and *β*1 and *β*2 represent the coefficients of *Z*_*t*_ and *K*_*t*_, respectively. The representation of the other symbols is the same as that of the single contamination model. All the analyses were performed with R software (R DevelopmentCore Team, 2015).

## 3. Results

### 3.1. Descriptive Analysis

**Figure 1:**
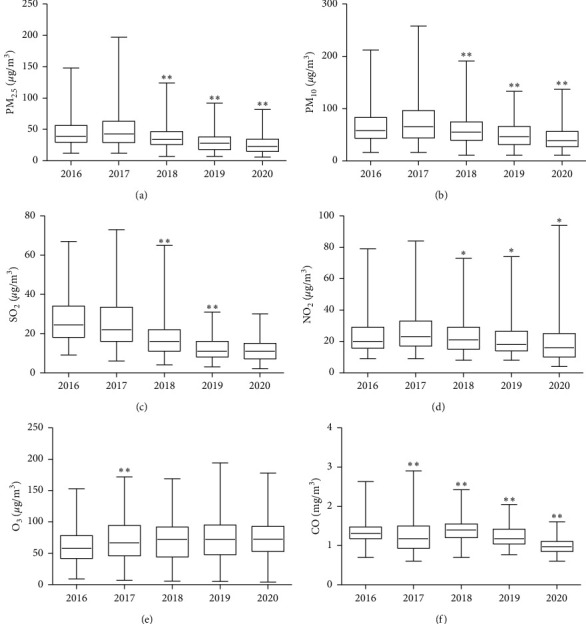
Box plot of the annual air pollution concentration in Ganzhou City, 2016–2020: (a) annual distribution of PM_2.5_ concentrations in 2016–2020, (b) annual distribution of PM_10_ concentrations in 2016–2020, (c) annual distribution of SO_2_ concentrations in 2016–2020, (d) annual distribution of NO_2_ concentrations in 2016–2020, (e) annual distribution of O_3_ concentrations in 2016–2020, and (f) annual distribution of CO concentrations in 2016–2020. “*∗*” indicates a difference (0.001 ≤ *P* < 0.05) between the contaminant concentration in the marker year and the previous year comparison. “∗∗” indicates more pronounced differences (*P* < 0.001) in pollutant concentrations in the marker year than in the previous year.

Table 1 describes the number, gender, and age distribution of hypertensive inpatients in the First Affiliated Hospital of Gannan Medical College during 2016–2020. We collected information on 2610 hospitalized patients with hypertension, of whom 52.68% were male and 39.62% were older than 65 years.  Table 2 describes the air pollution concentrations and meteorological parameters from 2016 to 2020 in Ganzhou City, China. The mean concentrations of CO, NO_2_, O_3_, PM_10_, PM_2.5_, and SO_2_ were 1.24, 23.51, 70.05, 60.11, 37.45, and 20.94 *μ*g/m^3^. The average temperature and relative humidity were 19.72°C and 74.37%, respectively, under a comfortable climate.  Figure 1 shows a box plot of the annual air pollution concentrations in 2016–2020, presenting the annual changes in air pollution concentrations. The concentrations of PM_2.5_, PM_10_, NO_2_, and CO showed an evident downward trend year by year from 2018 to 2020. The concentration of SO_2_ decreased significantly in 2018 and 2019, but the concentration of O_3_ was not observed. The differences in the six categories of air pollutants in different years are shown in Supplementary Table 1.

### 3.2. Spearman Correlation

Table 3 shows the Spearman correlation coefficient between air pollutants and meteorological conditions in Ganzhou during the study period. The rank correlation coefficients of all pairwise variables were significant (*P* < 0.001). The correlation analysis results among air pollutants show that except for the weak negative correlation between O_3_ and CO or NO_2_, all the other variables are positively correlated. A strong rank correlation is found between PM_2.5_ and PM_10_ with a correlation coefficient of 0.958. They are strongly correlated with NO_2_ and SO_2_, respectively. The correlation coefficient between air temperature and SO_2_ is 0.066, and the correlation coefficient between relative humidity and NO_2_ is −0.071, which can be regarded as no correlation. Temperature is negatively correlated with CO, NO_2_, PM_10_, PM_2.5_, and relative humidity and positively correlated with O_3_. The relative humidity is negatively correlated with O_3_, PM_10_, PM_2.5_, and SO_2_ and positively correlated with CO.

### 3.3. Analysis of Generalized Linear Model

**Figure 2:**
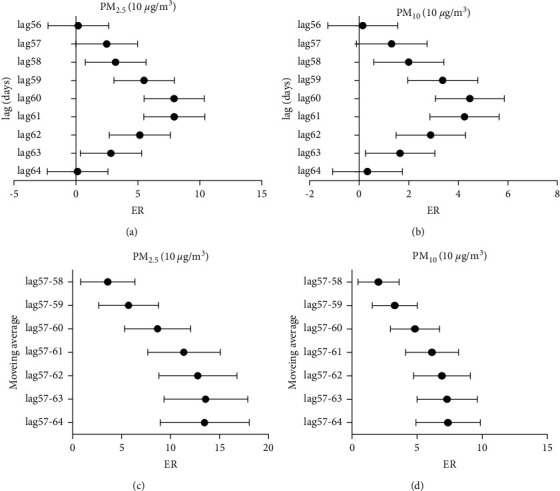
Excess risk (ER) and 95% confidence interval (CI) in daily hypertension hospital admissions per 10 *μ*g/m^3^ increase in PM_10_ and PM_2.5_ at different lag days: (a) excess risk (ER) in daily hypertension hospital admissions per 10 *μ*g/m^3^ increase in PM_2.5_, in single-day lag 56–64 days; (b) excess risk (ER) in daily hypertension hospital admissions per 10 *μ*g/m^3^ increase in PM_10_, in single-day lag 56–64 days; (c) excess risk (ER) in daily hypertension hospital admissions per 10 *μ*g/m^3^ increase in PM_2.5_, in moving average since 57 days; and (d) excess risk (ER) in daily hypertension hospital admissions per 10 *μ*g/m^3^ increase in PM_10_, in moving average since 57 days.

After calculation, PM_2.5_ and PM_10_ have a significant impact on the number of hypertension in patients with a daily lag of approximately 2 months.  Figure 2 shows the increase in the number of hospitalizations caused by an increase of 10 *μ*g/m^3^ for the atmospheric particulate matter at different lag periods when it is significantly correlated with daily hospitalizations for hypertension. We observed similar lag patterns for the effects of PM_2.5_ and PM_10_, except for lag day 57. For the single-day lag patterns, lag days 60 and 61 generated the highest estimates for the ambient fine particulate pollution. For a 10 *μ*g/m^3^ increase in PM_2.5_ and PM_10_ concentrations in the single-pollutant model, we observed significant increments in hospital admission on the lag day 60 at 7.91% (95% confidence interval: 5.50% to 10.37%) and 4.46% (95% confidence interval: 3.08% to 5.86%) and on the lag day 61 at 7.92% (95% confidence interval: 5.48% to 10.42%) and 4.24% (95% confidence interval: 2.86% to 5.65%).

The effects of moving average lags were higher than that of single lags in different periods [17]. According to the calculated significant single-day lag days, we calculated the daily moving average effect from 57 to 64 days to understand the significant impact of the single-day lag period, and the percentage of the change in the number of hospitalizations with the multiday moving average concentration of PM_2.5_ and PM_10_ increased by 10 *μ*g/m^3^. We evaluated the impact of environmental fine particulate pollution on the risk of hypertension hospitalization by considering the moving average effect during this period given that the continuous single-day lag effect was significant. In a moving mean effect with a lag of 57 to 64 days, an increase of 10 *μ*g/m^3^ in PM_2.5_ or PM_10_ concentrations was associated with a 13.41% (95% CI: 8.95% to 18.04%) or 7.29% (95% CI: 5.00% to 9.63%) increase in hospitalizations for hypertension patients, respectively.

**Figure 3:**
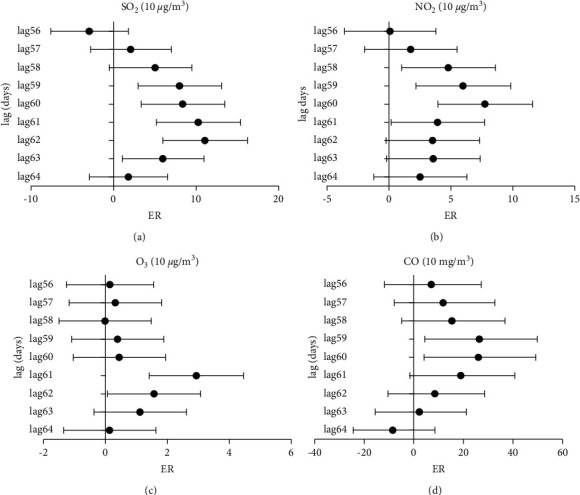
Excess risk (ER) and 95% confidence interval (CI) in daily hypertension hospital admission changes in the concentration of gaseous pollutants at different lag days: (a) excess risk (ER) in daily hypertension hospital admissions per 10 *μ*g/m^3^ increase in SO_2_, in single-day lag 56–64 days; (b) excess risk (ER) in daily hypertension hospital admissions per 10 *μ*g/m^3^ increase in NO_2_, in single-day lag 56–64 days; (c) excess risk (ER) in daily hypertension hospital admissions per 10 *μ*g/m^3^ increase in O_3_, in single-day lag 56–64 days; and (d) excess risk (ER) in daily hypertension hospital admissions per 10 mg/m^3^ increase in CO, in single-day lag 56–64 days.

The effects of other common air pollutants on hypertension hospital admissions were estimated in the period when atmospheric fine particulate pollution significantly affected the number of hypertension hospital admissions due to the significant correlation among these common air pollutants.  Figure 3 shows that NO_2_, O_3_, CO, and SO_2_ are significantly correlated with the number of hypertension hospital admissions at different degrees after 56 to 64 days of daily lag. For each increase in 10 *μ*g/m^3^ in NO_2_, O_3_, and SO_2_ levels, hypertension hospital admissions increased by 7.70% (95% CI: 3.95% to 11.59%) at lag 60 days, 2.93% (95% CI: 1.42% to 4.46%) at lag 61 days, and 10.99% (95% CI: 5.98% to 16.23%) at lag 62 days with the greatest impact. For each increase of 1 mg/m^3^ in the CO levels, hypertension hospital admissions increased by 2.93% (95% CI: 1.42% to 4.46%) at lag 61 days with the greatest impact. Among the four gaseous pollutants, SO_2_ had the largest number of days with significant influence on hypertension hospital admissions, ranging from lag 58 days to lag 63 days.

### 3.4. Sensitivity Analysis

We sequentially varied the df to control for secular trends in time, temperature, and relative humidity, thereby exploring the impact of changes in df from time and meteorological factors on the model fit results [18]. As shown in Table 4, when changing the df from long-term fluctuating trends of temporal and meteorological factors in the control model calculated at lag day 60, the results of PM_2.5_ concentration changes. As a result, the number of hospitalizations for hypertension showed only a small range of quantitative changes and did not affect the significance of the results. Therefore, the reliability of the model can be judged.

**Figure 4:**
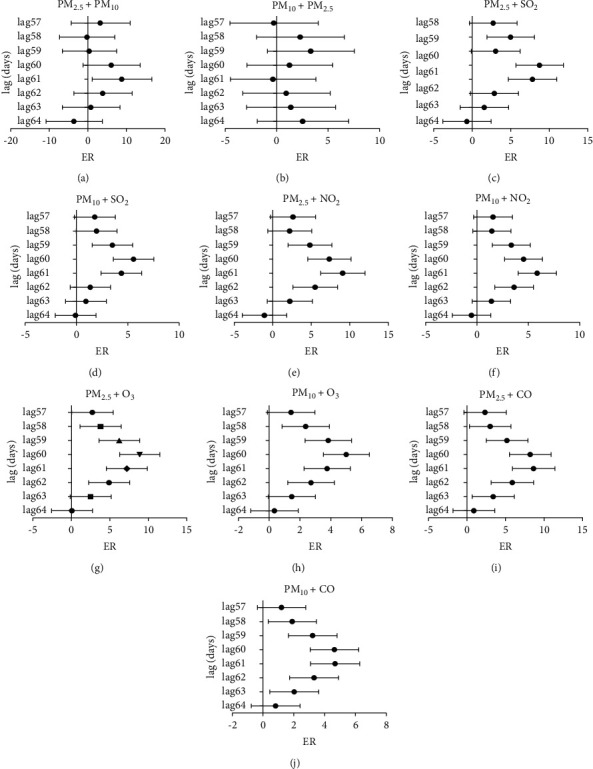
Excess risk (ER) with 95% confidence intervals in daily hypertension hospital admissions associated with 10 *μ*g/m^3^ increase in PM_2.5_ and PM_10_ concentrations in two-pollutant models: (a) after adjusting for PM_10_, excess risk (ER) in daily hypertension hospital admissions associated with per 10 *μ*g/m^3^ increase in PM_2.5_ concentrations in a lag of 57–64 single day; (b) after adjusting for PM_2.5_, excess risk (ER) in daily hypertension hospital admissions associated with per 10 *μ*g/m^3^ increase in PM_10_ concentrations in a lag of 57–64 single day; (c) after adjusting for SO_2_, excess risk (ER) in daily hypertension hospital admissions associated with per 10 *μ*g/m^3^ increase in PM_2.5_ concentrations in a lag of 57–64 single day; (d) after adjusting for SO_2_, excess risk (ER) in daily hypertension hospital admissions associated with per 10 *μ*g/m^3^ increase in PM_10_ concentrations in a lag of 57–64 single day; (e) after adjusting for NO_2_, excess risk (ER) in daily hypertension hospital admissions associated with per 10 *μ*g/m^3^ increase in PM_2.5_ concentrations in a lag of 57–64 single day; (f) after adjusting for NO_2_, excess risk (ER) in daily hypertension hospital admissions associated with per 10 *μ*g/m^3^ increase in PM_10_ concentrations in a lag of 57–64 single day; (g) after adjusting for O_3_, excess risk (ER) in daily hypertension hospital admissions associated with per 10 *μ*g/m^3^ increase in PM_2.5_ concentrations in a lag of 57–64 single day; (h) after adjusting for O_3_, excess risk (ER) in daily hypertension hospital admissions associated with per 10 *μ*g/m^3^ increase in PM_10_ concentrations in a lag of 57–64 single day; (i) after adjusting for CO, excess risk (ER) in daily hypertension hospital admissions associated with per 10 *μ*g/m^3^ increase in PM_2.5_ concentrations in a lag of 57–64 single day; and (g) after adjusting for CO, excess risk (ER) in daily hypertension hospital admissions associated with per 10 *μ*g/m^3^ increase in PM_10_ concentrations in a lag of 57–64 single day.

Significant effects on the number of hospitalizations for hypertension were present for other gaseous pollutants at similar lag periods when the effects of particulate matter on the number of hospitalizations for hypertension were significant. Thus, we used a two-pollutant model to identify whether the effects of particulate matter remained robust after correcting the effects of other pollutants.  Figure 4 shows that after adjustment of other air pollutants, the number of days, on which PM_2.5_ significantly affected those hospitalized with hypertension, decreased to varying degrees, but the association increased. After adjusting PM_10_, PM_2.5_ showed a significant association only at day 61 after the delay. We observed that PM_2.5_ showed the strongest effects at lag day 60 after adjusting the effects of NO_2_ and CO and the strongest impact effects at lag day 61 after adjusting the effects of SO_2_ and O_3_.

Figure 4 shows that when the effect of PM_2.5_ is added, PM_10_ has no effect on hypertension hospital admissions in the period when the 1 day lag has a significant effect. In the inclusion of CO or O_3_ in the double-pollutant model, the effects of PM_10_ are in line with that of the single-pollutant model. However, the associations between every 10 *μ*g/m^3^ increase in PM_10_ and lag day 61 were enhanced to 5.84% (95% CI: 3.99% to 7.73%) after adjusting the NO_2_.

## 4. Discussion

This study investigated the association between ambient particulate matter (PM_2.5_ and PM_10_) exposure and hypertension hospital admissions in Ganzhou, China, during the years 2016–2020, using a time-series model. The findings showed that exposures of PM_2.5_ and PM_10_ were positively associated with increased risk of hypertension hospital admissions. Thus far, this study is the first to investigate the adverse effects of air pollutants on hypertension in Ganzhou.

Since 2017, Ganzhou City intensified air pollution treatment action. With the exception of O_3_, an annual decreasing trend was observed in the concentrations of air pollutants, of which PM_2.5_ and PM_10_ were especially evident. Particulate matter concentrations were significantly negatively correlated with meteorological factors, probably because residents experience more combustion and energy-consuming heating behavior during the cold season. In addition, low temperatures in winter may contribute to poor dispersion of contaminants in the air, ultimately leading to peaks in winter contaminants [19]. Compared with most provincial capitals in southern China, the population density of Ganzhou is relatively sparse. However, with the reform and opening up and economic development, the real estate development in Ganzhou gradually rises in many places in recent years, and the number of motor vehicles grows rapidly. Construction dust, automobile exhaust, and manufacturing emissions may be the main sources of air pollution in Ganzhou City. The survey results show that in Ganzhou, the daily average concentrations of PM_10_ from 2016 to 2020 was 60.11 *μ*g/m^3^, which was higher than the national Ambient Air Quality Standard (GB3095-2012) primary standard but lower than the secondary standard (primary standard is 40 *μ*g/m^3^, and the secondary is 70 *μ*g/m^3^). The mean daily average concentrations of PM_2.5_ from 2016 to 2020 was 37.45 *μ*g/m^3^, which was higher than the NAQS secondary (primary standard is 15 *μ*g/m^3^, and the secondary is 35 *μ*g/m^3^).

Numerous epidemiological studies have investigated the effects of short- and long-term exposure to ambient air pollution on hypertension and blood pressure. However, the results were controversial. Results of a meta-analysis examining the associations between ambient air pollutants and blood pressure among children and adolescents showed that an increased risk of hypertension was associated with long-term PM_10_ exposure. In addition, long-term exposure to PM_2.5_ and PM_10_ significantly increases systolic and diastolic blood pressure. However, only PM_10_ showed a significant effect on blood pressure during the short-term exposure [20]. In a case-cross study that analyzed short-term effects of air pollutants on hospitalization rate in patients with cardiovascular disease, only CO was associated with an increased risk of admission in patients with hypertension. No clear evidence was found for short-term exacerbating effects for PM_2.5_ or PM_10_ [21]. We also calculated the short-term effects of changes in air pollution concentrations on hospitalized patients with hypertension on that day or lagged by 1 month in our study. However, we did not find statistically significant associated effects, as shown in Supplementary Table 2. The air pollution concentration was found to have a statistically significant positive result on the number of hospitalized patients with hypertension after two-month lag during calculation. Thus, we continued to collect the air pollution concentration data of Ganzhou City in October and November 2015. The effect of ambient particulate matter on hypertension in this study shows a medium- to long-term effect. The moving average effect of air pollution has maintained a significant effect on the number of hypertension hospital admissions long after a significant 1 day lag effect. Different from other studies, PM_2.5_ has a higher risk of excess hospitalization for hypertension patients, and the influence effect of PM_10_ is similar to that of most studies [20–22].

We also calculated the effect of air temperature on the number of hospitalizations for hypertension after adjusting the relative humidity, week effect, and time trend; the results are shown in Supplementary Figure 1. We found that air temperature variation had a short-term acute effect on the number of hospitalizations for hypertension. A single-day reduction in temperature significantly affects hospital admissions for hypertension on that day and after 1–3 days, with the greatest impact on hospital admissions after that day. The moving average change in temperature was calculated to affect the number of hospitalizations for hypertension in the lag day 10 days. Air temperature has an inverse correlation with particulate matter concentration, and reduced air temperature acutely increases hospital visits for hypertension in the short term. Thus, we speculate that the observed short-term effect of particulate matter concentration on disease may have resulted from the presence of collinearity between air temperature and particulate matter in the model. In addition, in cold environments, populations may reduce the behavior, such as outgoing or fenestration ventilation, which is less affected by outdoor pollution concentrations. Therefore, the effect of changes in PM_2.5_ and PM_10_ concentrations on the number of hospitalizations for hypertension during low temperature and high exposure may be influenced by confounding factors, such as the life habits of the population [23].

In addition to environmental fine particles, we calculated the effects of four other common air pollutants on hypertension and found that NO_2_, O_3_, CO, and SO_2_ showed varying degrees of impact at similar times. Studies have confirmed the effect of gaseous pollutants on aggravating hypertension, but the results in different regions are different [24–26].

We established a two-pollutant model to explore the changes in the level of impact of PM_2.5_ and PM_10_ on hypertension after adjusting the other pollutants due to the significant positive correlation between air pollutants. After adjustment of the second pollutant, the effective daily lag days of PM_2.5_ and PM_10_ decreased, but the excess risk peak of hypertension increased. The calculation results of the dual pollution model of PM_2.5_ and PM_10_ show that PM_2.5_ is only significantly correlated with hypertension hospital admissions at lag day 61, whereas PM_10_ has no significant effect. These results indicate that environmental fine particles are more likely to affect the number of hypertension hospital admissions 2 months later than gaseous pollutants. Compared with the two types of ambient fine particles, PM_2.5_ is a more important air pollution factor for hypertensive hospitalizations.

Current epidemiological studies on air pollution and hypertension mainly include long- and short-term exposure, and the distribution of disease is different. The final results presented are also different. The results of meta-analyses showed that hypertension was significantly associated with short-term exposure to SO_2_ (OR = 1.046, 95% CI: 1.012 to 1.081), PM_2.5_ (OR = 1.069, 95% CI: 1.003 to 1.141), and PM_10_ (OR = 1.024, 95% CI: 1.017 to 1.030) in each 10 *μ*g/m^3^ increment. In addition, a statistically significant increase was found in hypertension risk in association with each 10 *μ*g/m^3^ increment in the long-term exposure to NO_2_ (OR = 1.034, 95% CI: 1.005 to 1.063) and PM_10_ (OR = 1.034, 95% CI: 1.005 to 1.063) [11]. Air pollution was a short-term exposure, but it had a robust and significant correlation with hypertension hospital admissions with a lag of 2 months. The harmful components in the air pollution should experience a long period of metabolism and toxicological mechanism to cause harm to people with hypertension in Ganzhou City. We also found that after a significant impact of the 1 day lag, the moving average effect of PM_2.5_ and PM_10_ is associated with long-term stable hypertension hospital admissions. This finding also shows that the toxicological effects of air pollution may have a long-term mechanism expression process.

Several research teams have been carried out to study the mechanisms of particulate matter damage to the cardiovascular system, and there is currently evidence that atmospheric particulate matter can cause cardiovascular damage through oxidative stress, inflammatory responses, and DNA methylation pathways [27,28]. Metal elements (e.g., transition metals nickel, zinc) in PM_2.5_ components can induce redox imbalance caused by excessive reactive oxygen species (ROS) generation, leading to collective oxidative stress [29,30]. Superoxide dismutase (SOD), as a biomarker of oxidative stress, was observed to express obviously decreased in PM_2.5_-exposed experiments [31]. In the HUVEC model, PM_2.5_ exposure induced upregulation of IL-6 expression and decreased expression and nuclear localization of hypoxia-inducible factor-1 *α* at the transcriptional level, leading to inflammatory response and changes in integrity and permeability of vascular endothelial cell membrane [32]. A study found that short-term exposure to PM_2.5_, increased levels of angiotensin-converting enzyme (ACE), and decreased DNA methylation of the ACE gene were associated with increased blood pressure, which considered that hypomethylation of a specific ACE gene might be one of the main epigenetic mechanisms in PM_2.5_-raised hypertension [33].

There are still some problems in our research. First of all, the air pollutant exposure data we collected are all obtained from five fixed air monitoring points in Ganzhou city center, and only a few fixed points of environmental exposure dose are measured. It is difficult to quantify the specific biological effective dose of human exposure. This error may lead our research to misjudge the impact of air pollutants. Secondly, our data source was only from a large general hospital in Ganzhou City, whose daily number of hypertension inpatients was limited, so it was not suitable for subgroup analysis. This may lead to our neglect of the impact of air pollution on hypertension patients, which may have individual differences such as gender, age, and obesity [34]. Third, only other pollutants, time trend, temperature, relative humidity, and weekend effect are balanced in the model, while other influencing factors such as legal holidays and influenza pandemic variables are not included in the model. For example, at the beginning of 2020, due to the outbreak of COVID-19, the number of hospitalized patients decreased significantly compared with the same period. This kind of phenomenon exists due to the reduction of human outgoing activities during this period, which reduces urban air pollution and consequently reduces the harm to cardiovascular diseases, and there are also reasons such as the reduction in the number of hospitalizations due to restricted movement of people [35].

## 5. Conclusions

We found that PM_2.5_ and PM_10_ exposure has a mid- to long-term impact on the risk of hospitalization due to hypertension after a lag of nearly 2 months in Ganzhou City, Jiangxi Province, China. PM_2.5_ is the main influencing factor in the exposure of various air pollutants. These findings may help better understand the impact of environmental particulates on health.

## Figures and Tables

**Table 1:** Distribution of hospitalized patients with hypertension (ICD-10: I10–I13) and its subgroups during 2016–2020.

	2016–2020	2016	2017	2018	2019	2020
Total	2,610 (100%)	464 (17.78%)	423 (16.21%)	619 (23.72%)	596 (22.84%)	508 (19.46%)
Male	1,375 (52.68%)	240 (9.20%)	221 (8.47%)	308 (11.80%)	333 (12.76%)	273 (10.46%)
Female	1,235 (47.32%)	224 (8.58%)	202 (7.74%)	311 (11.92%)	263 (10.08%)	235 (9.00%)
Age >65	1,034 (39.62%)	202 (7.74%)	169 (6.48%)	261 (10.00%)	211 (8.08%)	191 (7.32%)
Age <65	1,576 (60.38%)	262 (10.04%)	254 (9.73%)	358 (13.72%)	385 (14.75%)	317 (12.15%)

**Table 2:** Descriptive summary of daily air pollutants and meteorological factors during 2016–2020 in Ganzhou, China.

Variable	Minimum	P25	Median	P75	Maximum	Mean (SD)
Air pollutant^a^						
CO (mg/m³)	0.60	1	1.2	1.44	2.9	1.24 (0.32)
NO_2_ (*μ*g/m³)	4	14	20	28	94	23.51 (13.59)
O_3_ (*μ*g/m³)	4	46	67	91	194	70.05 (32.63)
PM_10_ (*μ*g/m³)	11	36	52	76	258	60.11 (33.91)
PM_2.5_ (*μ*g/m³)	6	23	33	47	197	37.45 (21.20)
SO_2_ (*μ*g/m³)	2	11	16	23	73	18.74 (11.26)
Weather						
Temp (°C)	0	13	21	27	32	19.72 (8.07)
HR (%)	35	65	74	84	99	74.37 (12.19)

^a^Average of air pollution concentration measured at five monitoring points in the central city of Ganzhou.

**Table 3:** Spearman's correlation between air pollutants and meteorological factors in Ganzhou, China, during 2015–2020.

	CO	NO_2_	O_3_	PM_10_	PM_2.5_	SO_2_	Temp
NO_2_	0.401^*∗∗*^						
O_3_	−0.182^*∗∗*^	−0.095^*∗∗*^					
PM_10_	0.386^*∗∗*^	0.655^*∗∗*^	0.327^*∗∗*^				
PM_2.5_	0.445^*∗∗*^	0.618^*∗∗*^	0.257^*∗∗*^	0.958^*∗∗*^			
SO_2_	0.292^*∗∗*^	0.510^*∗∗*^	0.220^*∗∗*^	0.705^*∗∗*^	0.687^*∗∗*^		
Temp	−0.320^*∗∗*^	−0.394^*∗∗*^	0.370^*∗∗*^	−0.126^*∗∗*^	−0.211^*∗∗*^	0.066^*∗∗*^	
HR	0.210^*∗*^	−0.073^*∗∗*^	−0.642^*∗∗*^	−0.359^*∗∗*^	−0.228^*∗∗*^	−0.293^*∗∗*^	−0.316^*∗∗*^

^*∗∗*^ *P* < 0.001.

**Table 4:** Changes in PM_2.5_ concentration on lag day 60 and changes in 95% CIs excess risk (ER) of hypertension hospital admissions with different df values of time, temperature, and relative humidity.

Variable	df	AIC	ER	95% CI
Time	6	5,509.46	7.51	5.11–9.97
	7^*∗*^	5,501.516	7.90	5.50–10.36
	8	5,485.721	7.81	5.41–10.26
	5	5,499.726	7.89	5.49–10.35

Temperature	6^*∗*^	5,501.516	7.90	5.50–10.36
	7	5,501.936	7.82	5.42–10.28
	2	5,499.51	7.92	5.52–10.37

Relative humidity	3^*∗*^	5,501.516	7.90	5.50–10.36
	4	5,503.707	7.89	5.49–10.35

## Data Availability

The data for the research were obtained from the Big Data Center of the First Affiliated Hospital of Gannan Medical University and the National Urban Air Quality Real-Time Publishing Platform containing details held by detailed admission time and diagnosis of cardiovascular patients. The authors elect not to share data.

## Supplementary Materials

Supplementary Materials Supplementary Table 1: Results of a comparison test of the mean value of air pollution concentration in Ganzhou City during 2016–2020. Supplementary Table 2: Varying percentages of different lag days air pollution concentrations and daily number of hospitalizations for hypertension under single-pollutant models. Supplementary Figure 1: Excess risk (ER) and 95% confidence interval (CI) in daily hypertension hospital admissions per 1℃ increase in temperature at different lag days.
