# Supplementary material for: Association between Particulate Matter Pollution Concentration and Hospital Admissions for Hypertension in Ganzhou, China
Source: Int J Hypertens. 2022 Feb 17;2022:7413115. doi: 10.1155/2022/7413115 (PMC8872648; doi:10.1155/2022/7413115)
Supplement: Supplementary Materials — Supplementary Table 1: Results of a comparison test of the mean value of air pollution concentration in Ganzhou City during 2016–2020. Supplementary Table 2: Varying percentages of different lag days air pollution concentrations and daily number of hospitalizations for hypertension under single-pollutant models. Supplementary Figure 1: Excess risk (ER) and 95% confidence interval (CI) in daily hypertension hospital admissions per 1℃ increase in temperature at different lag days. [file 7413115.f1.doc]

**Supplementary tables and figures**

**Supplementary Table 1 Results of comparison test of mean value of air pollution concentration in Ganzhou City during 2016-2020.**

| Comparison years | CO | NO2 | O3 | PM10 | PM2.5 | SO2 |
| --- | --- | --- | --- | --- | --- | --- |
| 2016-2017 | 0.000 | 0.052 | 0.001 | 0.679 | 1.000 | 0.348 |
| 2016-2018 | 0.470 | 1.000 | 0.002 | 0.159 | 0.001 | 0.000 |
| 2016-2019 | 0.000 | 0.011 | 0.000 | 0.000 | 0.000 | 0.000 |
| 2016-2020 | 0.000 | 0.000 | 0.000 | 0.000 | 0.000 | 0.000 |
| 2017-2018 | 0.000 | 0.020 | 1.000 | 0.000 | 0.000 | 0.000 |
| 2017-2019 | 1.000 | 0.000 | 1.000 | 0.000 | 0.000 | 0.000 |
| 2017-2020 | 0.000 | 0.000 | 1.000 | 0.000 | 0.000 | 0.000 |
| 2018-2019 | 0.000 | 0.030 | 1.000 | 0.000 | 0.000 | 0.000 |
| 2018-2020 | 0.000 | 0.000 | 0.958 | 0.000 | 0.000 | 0.000 |
| 2019-2020 | 0.000 | 0.023 | 1.000 | 0.001 | 0.020 | 1.000 |

**Supplementary Table 2 Varying percentages of different lag days air pollution concentrations and daily number of hospitalizations for hypertension under single pollutant models.**

| Single day lag | PM2.5 | PM10 | SO2 | NO2 | O3 | CO |
| --- | --- | --- | --- | --- | --- | --- |
| Lag0 | 2.00(-0.73to4.81) | 1.28(-0.36to 2.94) | 3.08(-2.45to 8.93) | 2.50(-1.14to 6.27) | 0.57(-1.42to 2.61) | 1.06(-1.08to 3.24) |
| Lag1 | 1.46(-1.22to4.21) | 0.88(-0.71to 2.50) | 0.59(-4.51to 5.96) | 2.45(-1.28to 6.33) | -0.21(-1.98to 1.59) | 1.62(-0.49to 3.77) |
| Lag2 | -1.18(-3.73to1.44) | -0.84(-2.36to 0.69) | -1.49(-6.41to 3.70) | -2.55(-6.16to 1.19) | -0.16(-1.75to 1.44) | 0.07(-1.91to 2.09) |
| Lag3 | -2.81(-5.29to-0.27) | -1.98(-3.45to-0.49) | -5.42(-10.12to -0.47) | -4.37(-7.91to -0.70) | -1.92(-3.42to -0.40) | -0.46(-2.37to 1.49) |
| Lag4 | 0.09(-2.36to2.60) | -0.52(-1.96to 0.93) | -1.47(-6.26to 3.56) | -3.44(-6.90to 0.14) | -1.72(-3.20to -0.23) | 0.86(-1.04to 2.79) |
| Lag5 | 1.01(-1.43to3.52) | 0.06(-1.35to 1.50) | -1.56(-6.31to 3.44) | -2.76(-6.19to 0.80) | -1.18(-2.66to 0.32) | -0.09(-1.97to 1.83) |
| Lag6 | 1.51(-0.93to4.00) | 0.38(-1.02to 1.80) | 2.15(-2.69to 7.22) | -2.10(-5.55to 1.48) | -0.76(-2.23to 0.73) | 0.32(-1.57to 2.25) |
| Lag13 | 0.58(-1.86to 3.09) | -0.13(-1.54to 1.30) | 1.73(-3.06to 6.76) | 0.44(-3.05to 4.05) | -0.19(-1.67to 1.30) | -0.78(-2.66to 1.13) |
| Lag20 | -2.05(-4.43to 0.39) | -1.20(-2.58to 0.20) | -3.85(-8.41to 0.94) | -0.89(-4.36to 2.71) | -2.30(-3.76to -0.83) | -1.15(-3.01to 0.73) |
| Lag27 | 0.94(-1.47to 3.41) | 0.65(-0.73to 2.05) | -1.70(-6.31to 3.13) | 0.62(-2.87to 4.23) | -0.32(-1.77to 1.15) | 0.35(-1.52to 2.26) |

**
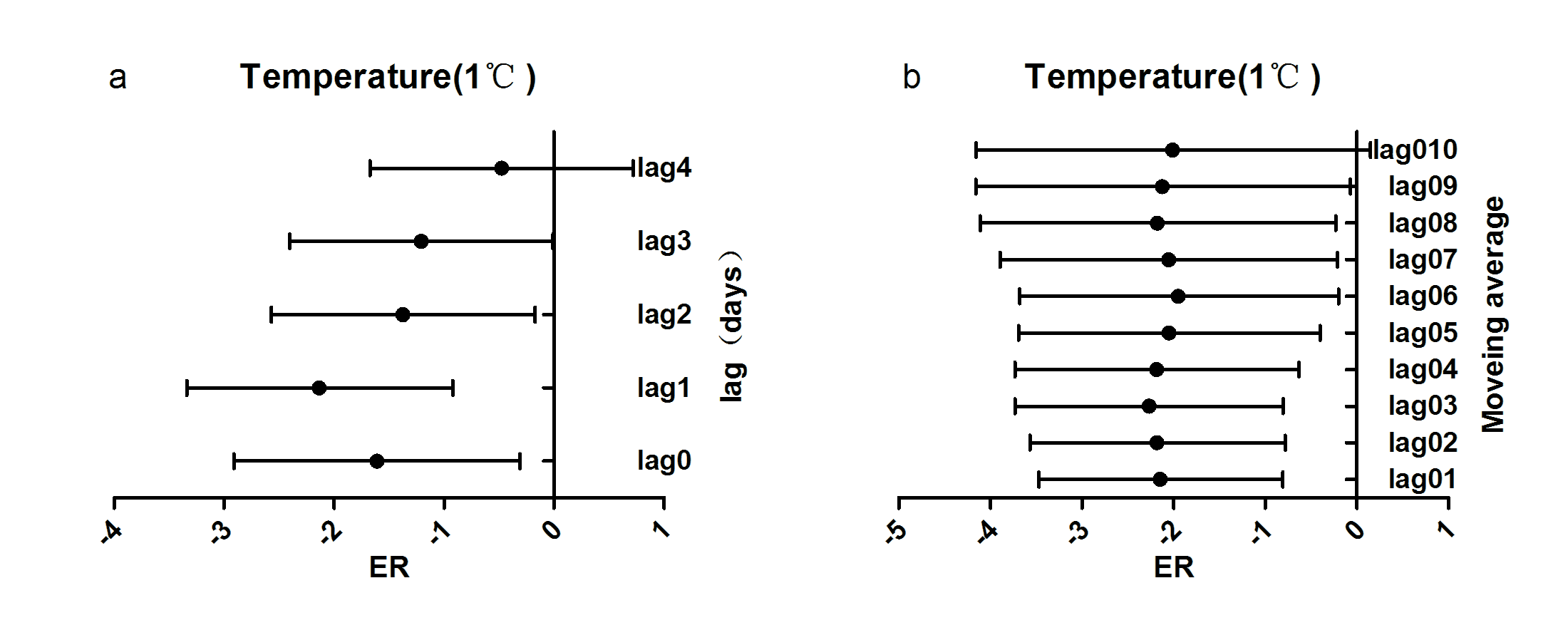
**

**Supplementary Figure 3. Excess risk (ER) and 95% confidence interval (CI) in daily hypertension hospital admissions per 1℃ increase in temperature increase at different lag days.** (a) Excess risk (ER) in daily hypertension hospital admissions per 1℃ increase in temperature increase in single day lag 0-4 days. (b) Excess risk (ER) in daily hypertension hospital admissions per 1℃ increase in temperature increase in moving average 1-10 days.
